# Outcomes of Deferoxamine Action on H_2_O_2_-Induced Growth Inhibition and Senescence Progression of Human Endometrial Stem Cells

**DOI:** 10.3390/ijms22116035

**Published:** 2021-06-03

**Authors:** Alla N. Shatrova, Elena B. Burova, Marianna V. Kharchenko, Irina S. Smirnova, Olga G. Lyublinskaya, Nikolay N. Nikolsky, Aleksandra V. Borodkina

**Affiliations:** Department of Intracellular Signaling and Transport, Institute of Cytology, Russian Academy of Sciences, Tikhoretsky Ave. 4, 194064 St. Petersburg, Russia; lenbur87@mail.ru (E.B.B.); mariasha2000@mail.ru (M.V.K.); sisfromspb@yahoo.com (I.S.S.); o.lyublinskaya@mail.ru (O.G.L.); cellbio@incras.ru (N.N.N.); borodkina618@gmail.com (A.V.B.)

**Keywords:** human endometrial stem cells, deferoxamine, antioxidants, oxidative stress, SIPS, HIF-1 α

## Abstract

Mesenchymal stem cells (MSCs) are broadly applied in regenerative therapy to replace cells that are lost or impaired during disease. The low survival rate of MSCs after transplantation is one of the major limitations heavily influencing the success of the therapy. Unfavorable microenvironments with inflammation and oxidative stress in the damaged regions contribute to MSCs loss. Most of the strategies developed to overcome this obstacle are aimed to prevent stress-induced apoptosis, with little attention paid to senescence—another common stress reaction of MSCs. Here, we proposed the strategy to prevent oxidative stress-induced senescence of human endometrial stem cells (hMESCs) based on deferoxamine (DFO) application. DFO prevented DNA damage and stress-induced senescence of hMESCs, as evidenced by reduced levels of reactive oxygen species, lipofuscin, cyclin D1, decreased SA-β-Gal activity, and improved mitochondrial function. Additionally, DFO caused accumulation of HIF-1α, which may contribute to the survival of H_2_O_2_-treated cells. Importantly, cells that escaped senescence due to DFO preconditioning preserved all the properties of the initial hMESCs. Therefore, once protecting cells from oxidative damage, DFO did not alter further hMESCs functioning. The data obtained may become the important prerequisite for development of a new strategy in regenerative therapy based on MSCs preconditioning using DFO.

## 1. Introduction

Mesenchymal stem cells (MSCs) are a unique type of cells in the adult organism that possess exceptional properties including self-renewal, differentiation, homing, immunomodulatory and anti-inflammatory effects [1]. Namely due to these properties, MSCs present an essential source for tissue engineering to repair damaged tissues and organs [1]. Many sources of MSCs have been discovered since 1968, when MSCs were first isolated from the bone marrow [2]. Adipose tissue, synovium and synovial fluid, dental pulp, skin, muscle and others are among the discovered MSCs sources [3]. Recently, MSCs were found in the endometrium, particularly in the desquamated endometrium contained in menstrual blood [4,5,6]. The obvious advantages of the latter are non-invasive and non-traumatic isolation procedure, along with the enhanced replicative potential of human endometrial stem cells (hMESCs) compared to MSCs from bone marrow and adipose tissue [6,7]. The low success rates after MSCs transplantation despite the origin still remains the unresolved challenge, as harsh microenvironments in the damaged regions (e.g., poor blood supply, low oxygen tension, and inflammation together generating oxidative stress) heavily influence the functioning and viability of the transplanted cells [8,9,10,11,12]. Huge efforts are performed to overcome this obstacle, focused primarily on preventing MSCs apoptosis induced by unfavorable microenvironments during transplantation [10,12,13]. However, apoptosis is not the only stress reaction of MSCs. Another important reaction of MSCs towards various types of stresses, including the oxidative one, is senescence [14,15,16]. In fact, MSCs have been proven to be highly resistant to apoptosis, while prone to stress-induced premature senescence (SIPS) [14,15,17]. According to the most accepted definition, senescent cells irreversibly lose proliferation capacity, but retain their viability and metabolic activity, which is, however, altered from that of their younger counterparts. Along with the irreversible growth arrest MSCs that underwent SIPS display all other classical features of senescence, including irreparable DNA damage, increased cell size, elevated reactive oxygen species levels (ROS), impaired mitochondrial, lysosomal, ribosomal functioning, senescence-associated β-galactosidase activity (SA-β-Gal), accumulation of lipofuscin, and altered secretory phenotype, termed senescence-associated secretory phenotype (SASP) [16,18,19]. Bearing in mind the recent evidence that beneficial effects of MSCs-based therapy are predominantly mediated via the paracrine action rather than the direct differentiation and substitution of damaged cells, acquisition of SASP during MSCs senescence may have extremely deleterious effects upon transplantation [20,21,22]. Thus, senescence of MSCs induced by stressful microenvironments during transplantation is on the one hand highly probable, and on the other hand a highly undesirable event that should be necessarily prevented.

The aim of the present study was to design a strategy to prevent oxidative stress-induced senescence of hMESCs that can be performed immediately before cell transplantation and will allow avoiding undesirable stress reaction of the transplanted cells. We selected a well-known and clinically approved iron chelator deferoxamine (DFO) as a possible “senescence-preventing” agent due to its protective role against H_2_O_2_-induced DNA damage that was demonstrated using different cell models [23,24,25]. The following concrete approaches were formulated to achieve the aim of the study: (i) to develop an optimal scheme for processing cells with DFO, which would lead to its maximum protective effect against the induction and progression of senescence and which would not alter further cell functioning; (ii) to uncover possible mechanisms of the protective role of DFO.

## 2. Results

### 2.1. Pretreatment with 1 mM DFO for 2 h Is Optimal to Protect hMESCs from the Oxidative Stress-Induced Growth Arrest

**Figure 1:**
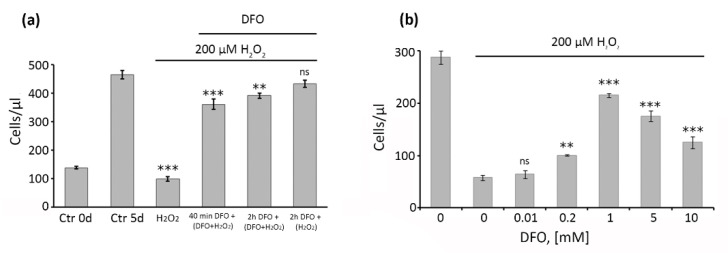
Pretreatment of hMESCs by DFO at concentration 1 mM for 2 h is optimal for protection of cells from oxidative stress-induced growth arrest. (**a**) Here we varied the time DFO pretreatment (40 and 120 min) and the mode (presence or absence of co-treatment with DFO and H_2_O_2_) of DFO exposure; DFO was used at a concentration of 1 mM; after oxidative stress cells were washed from the drugs and cultivated in the fresh growth medium for additional five days; FACS analysis was performed five days after stress has been completed; cells number were determined as the number of PI-negative (live) cells; (**b**) dose-dependent effect of DFO against H_2_O_2_-induced growth inhibition in hMESCs. Cells were pretreated with the indicated concentrations of DFO for 2 h, then washed from DFO and subjected to oxidative stress with 200 μM of H_2_O_2_ for 1 h; FACS was performed was performed five days after the oxidative stress. Data are shown as Mean ± SD, *n* = 3; *** *p* < 0.005, ** *p* < 0.01. Schemes follow the same formatting.

Previously, utilizing subcytotoxic treatments of cells with hydrogen peroxide (1 h 200 μM H_2_O_2_), we have developed and studied in detail the model SIPS of hMESCs [14,26]. Herein, we applied this model to test the protective effect of DFO. We first optimized DFO treatment conditions, namely duration and dose. To do so, we varied DFO treatment parameters and assessed the number of cells, since proliferation arrest is considered to be the most undesirable outcome of cell senescence. Being a large molecule, DFO enters cells through liquid-phase endocytosis, which requires relatively long-term cells exposure with the chelator [27,28,29,30]. Based on that, we selected the following DFO supplementation schemes: 40 min pretreatment plus constant presence during 1 h oxidative stress (indicated as 2 in Figure 1a); 2 h pretreatment plus constant presence during 1 h oxidative stress (indicated as 3 in Figure 1a); 2 h pretreatment without presence during 1 h oxidative stress (indicated as 4 in Figure 1a). In this set of the experiments DFO was used at the fixed concentration 1 mM. As expected, the number of viable hMESCs in 5 days after 1 h H_2_O_2_ treatment was comparable to that of the control cells at the day of the treatment, clearly indicating proliferation arrest (Figure 1a). Contrarily, the number of DFO pretreated hMESCs was significantly higher, with the most pronounced effect obtained using 2 h DFO pretreatment, demonstrating that such a treatment scheme is optimal for cell protection against H_2_O_2_-induced growth arrest (Figure 1a).

We next varied DFO concentration from 10 μM to 10 mM using 2 h pretreatment and assessed changes in cell proliferation. As shown in Figure 1b, the protective effect of DFO reached maximum at 1 mM concentration; while the lower doses were less effective in prevention of H_2_O_2_-induced growth arrest, the higher doses of DFO themselves slowed down hMESCs proliferation (without any cell death). Thus, 2 h pretreatment with 1mM DFO turned out to be the most optimal to protect hMESCs from the growth arrest caused by H_2_O_2_ and in the following experiments we routinely used it.

### 2.2. DFO Pretreatment Is Sufficient to Prevent Oxidative Stress-Induced Senescence of hMESCs

**Figure 2:**
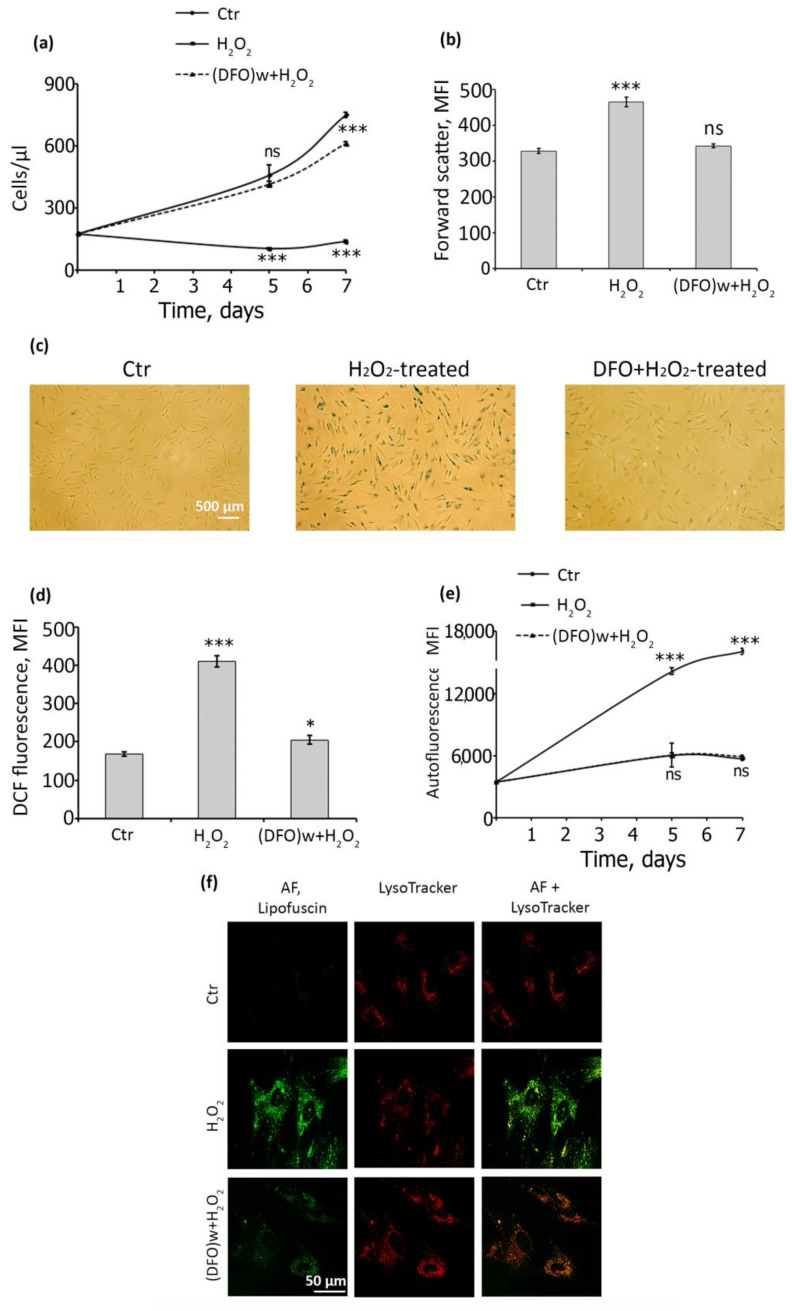
DFO pretreatment is sufficient to prevent H_2_O_2_-induced senescence of hMESCS. (**a**) Growth curves of the control, H_2_O_2_-treated and (DFO + H_2_O_2_)-treated cells estimated on the 5th and 7th days after the stress. (**b**) Forward scatter reflecting cell size analyzed on 5th day after the oxidative insult. (**c**) Representative microphotographs of SA-β-Gal staining. Scale bar 500 μm is valid for all images. (**d**) Intracellular ROS levels assessed by FACS using dichlorodihydrofluorescein diacetate (DCF) staining. (**e**) Lipofuscin accumulation estimated by AF measurement. Data are shown as Mean ± SD, *n* = 3; *** *p* < 0.005, * *p* < 0.05. (**f**) Effect of DFO on H_2_O_2_-induced accumulation of lipofuscin and its co-localization with lysosomal compartment in hMESCs. Cells were stained with the vital dye LysoTracker Red to detect lysosomes. To identify lipofuscin, which has a broad spectrum of autofluorescence, we used 510–550 nm excitations light to visualize cells in real time using confocal microscopy.

Having established the optimal DFO application parameters, we checked whether it could fully avert senescence of hMESCs by assessing a plethora of senescence-associated parameters. To begin with, we investigated the effect of DFO on the proliferation of H_2_O_2_-treated hMESCs during prolonged cultivation. As can be seen in Figure 2a, on the seventh day of cultivation, cells pretreated with DFO continued to proliferate, such as control cells, whereas cells treated with H_2_O_2_ alone (senescent) did not.

Additionally, DFO pretreatment completely abolished cell hypertrophy, i.e., the size of hMESCs pretreated with DFO was comparable with that of the control ones, while H_2_O_2_-treated cells were noticeably larger (Figure 2b). SA-β-Gal activity, another common marker of senescent cells, was almost undetectable in DFO-pretreated cells, contrarily to H_2_O_2_-treated hMESCs (Figure 2c). We next estimated the levels of intracellular reactive oxygen species (ROS), since senescent cells are generally characterized by the elevated endogenous ROS production [26]. Indeed, H_2_O_2_-treated hMESCs displayed more than twice-higher ROS levels compared either to control or to DFO-pretreated cells (Figure 2d). According to the literary data, there is a relationship between ROS production and lipofuscine (LF) accumulation during the development of the stress-induced senescence [31]. Thus, currently, LF accumulation that is estimated simply by the enhancement in cell autofluorescence is considered as a reliable in vitro marker of cellular senescence [32]. We also revealed a clear positive correlation between intracellular ROS elevation and LF accumulation during hMESCs senescence development (Supplementary Figure S1). Moreover, in H_2_O_2_-treated hMESCs the mean autofluorescence gradually increased during the whole observation period, while in DFO-pretreated cells it remained at the control level (Figure 2e). Additionally, we tested whether DFO attenuated LF accumulation within the lysosomal compartment. To do so, we used the vital dye LysoTracker Red and monitored the effect of DFO on stress-induced LF accumulation using confocal microscopy with live cell imaging. As can be seen in the images, LF granules were undetectable in control cells, but they were clearly visible in cells treated with H_2_O_2_, more specifically within the lysosomal compartment of the treated cells (Figure 2f). DFO, in turn, prevents stress-induced LF accumulation in the lysosomal compartment, as evidenced by the attenuation of LF fluorescence inside lysosomes (Figure 2f).

**Figure 3:**
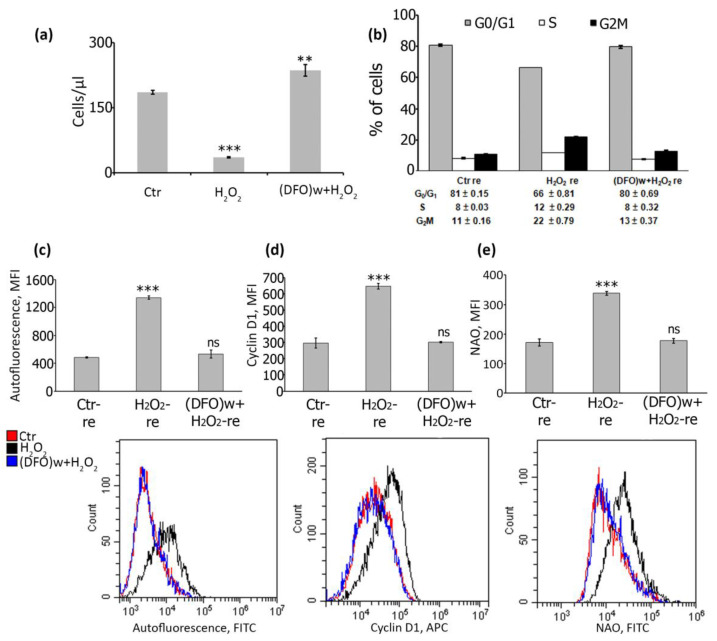
The protective effect of the single-dose DFO pretreatment against H_2_O_2_-induced senescence is preserved after cell reseeding. (**a**) The number of viable PI-negative cells determined in untreated, H_2_O_2_-treated and DFO-pretreated-hMESCs after reseeding. (**b**) Cell cycle distribution of the same cells. (**c**–**e**) FACS analysis of the senescence markers after cell reseeding: lipofuscin (**c**), cyclin D1 (**d**), mitochondrial mass as nonyl acridine orange (NAO) fluorescence (**e**) (histograms (top line) and the appropriate representative distributions (bottom line)). Data are shown as Mean ± SD, *n* = 3, *** *p* < 0.005, ** *p* < 0.01.

Next, we applied a cell reseeding technique to test the protective DFO action against oxidative stress during prolonged cultivation. We reseeded the control, H_2_O_2_-treated and DFO + H_2_O_2_-treated hMESCs five days after the oxidative insult, then cultured them for an extra three days and then analyzed various cell parameters related to senescence. Firstly, DFO-pretreated cells retained proliferative capacity after reseeding similar to the control ones, while H_2_O_2_-treated hMESCs remained arrested (Figure 3a). 

Analysis of cell cycle phase distribution correlated well with the above results–H_2_O_2_-treated cells were in the G_2_M block, and DFO-pretreated cells exhibit exactly the same cell cycle phase distribution as control hMESCs (Figure 3b). Similarly, the average AF intensity of cells exposed to H_2_O_2_ alone was almost three times higher than that of untreated or pretreated with DFO (Figure 3c). We further verified DFO-protective effect by estimation of the cyclin D1 levels and mitochondrial mass in reseeded cells, as accumulation of cyclin D1 and increase in mitochondrial mass are well-recognized markers of senescent cells [26,33]. Indeed, we observed a significant increase in fluorescence intensity in senescent hMESCs stained with anti-cyclin D1 antibodies compared to cells pretreated with DFO that had the same level of cyclin D1 as untreated control cells (Figure 3d). Therefore, DFO prevented cyclin D1 accumulation in hMESCs subjected to oxidative stress. Finally, we revealed a noticeable elevation in mitochondrial mass in senescent cells using nonyl acridine orange (NAO) staining, however in DFO-pretreated cells the mitochondrial mass value was indistinguishable from the control ones (Figure 3e). Together, the results presented within this part provide solid evidence of the protective ability of single-dose DFO pretreatment against progression of stress-induced senescence in hMESCs.

### 2.3. DFO-Rescued hMESCs Retain the Ability to Respond towards Oxidative Stress via Senescence Induction

**Figure 4:**
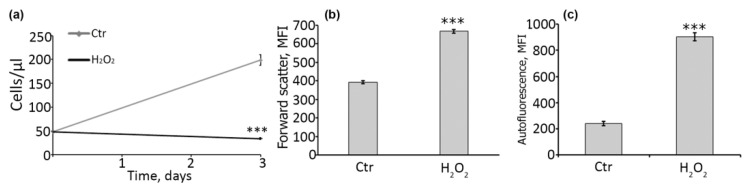
DFO-rescued hMESCs enter senescence-like state upon repeated oxidative stress. To test the stress reactions of H_2_O_2_-treated hMESCs that escaped senescence due to DFO pretreatment, such cells were seeded at equal density in 7 days after the initial treatment and either remained untreated (control cells) or were subjected to the repeated oxidative stress (200 µM of H_2_O_2_ for 1 h). Cells were cultivated for additional three days and analyzed by FACS. (**a**) Growth curves, (**b**) FSC (forward scattering), (**c**) AF (autofluorescence). Data are shown as Mean ± SD, *n* = 3, *** *p* < 0.005.

By its physiological meaning, senescence functions to prevent expansion of cells bearing damage and, therefore, is considered as the essential cell response against tumorigenesis [34]. The proper functioning of MSCs implies the ability to react on the damage by senescence initiation. Otherwise, such cells may become malignant. In this regard, hMESCs once escaped from the oxidative stress-induced senescence due to the protective effect of DFO should further restore this stress-reaction. The latter seems obligatory for the safety transplantation of DFO-pretreated hMESCs. Thus, we next investigated the fate of DFO-pretreated hMESCs upon the repeated oxidative insult. In brief, hMESCs, which were initially pretreated with DFO and then subjected to the first oxidative stress accordingly to the optimized protocol described above, in 7 days were stressed again (200 μM 1 h) and additionally cultured for 3 days before FACS analysis. Indeed, when subjected to the repeated oxidative stress the ‘progeny’ of DFO-rescued cells entered senescence-like state, as indicated by the proliferation loss, increased cell size and enhanced autofluorescence (Figure 4). To sum up, single-dose DFO pretreatment can be considered as a safe protocol to prevent oxidative damage just after hMESCs transplantation, without any alteration in the overall stress-responsiveness of the transplanted cells. 

### 2.4. DFO Protects DNA Damage by Inhibiting the Formation of Hydroxyl Radicals in H_2_O_2_-Treated hMESCs

**Figure 5:**
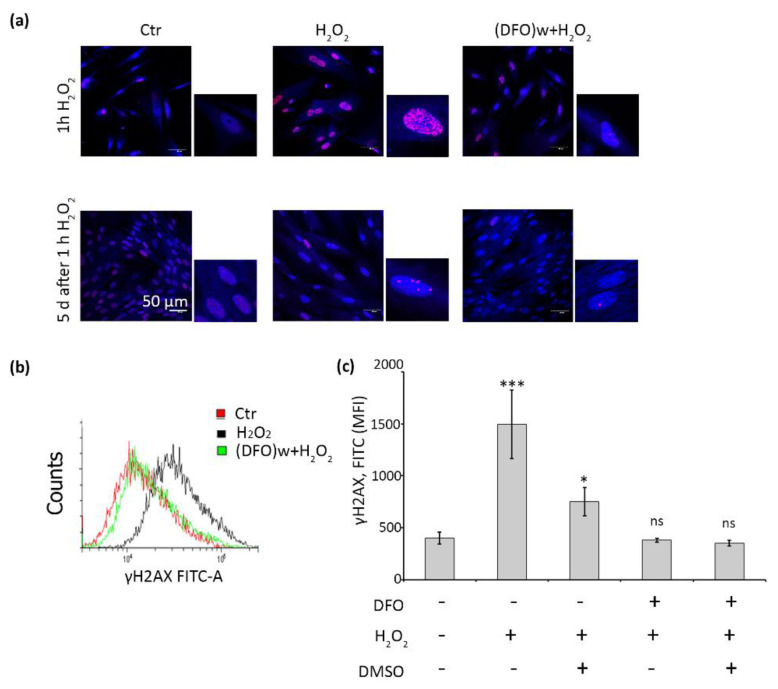
DFO prevents DNA damage and γH2AX foci formation in H_2_O_2_-stressed hMESCs. (**a**) Immunofluorescent analysis of γH2AX foci formation directly after stress execution and five days later; images are performed at 100× magnification, the scale bar is 50 µm, the enlarged insets are 50 × 50 µm; (**b**) representative histograms of the samples stained with γH2AX antibodies analyzed by FACS directly after stress execution; (**c**) to elucidate the role of hydroxyl radicals in DNA-oxidative damage of hMESCs DMSO was applied and γH2AX intensity was assessed by FACS. Data are shown as Mean ± SD, *n* = 3, * *p* < 0.05, *** *p* < 0.005.

Having established the fact of the protective DFO action against oxidative stress-induced senescence of hMESCs, we further tried to uncover intracellular processes related to its senescence-preventing effects. Stress-induced senescence of hMESCs is accompanied by the persistent DNA damage and DNA damage response (DDR) activation [26]. The latter is responsible for the irreversible proliferation loss during cell senescence. Herein we utilized antibodies recognizing phosphorylated histone γH2AX a well-known marker of double strand breaks to check the protective role of DFO against the oxidative DNA damage. Immunofluorescence analysis showed a large number of foci in H_2_O_2_-treated cells immediately after the stress induction, which persisted for the next five days that confirms a long-term activation of DDR in senescent hMESCs. On the contrary, only a small part of cells pretreated by DFO contained well-detected foci just after the oxidative stress (Figure 5a). Moreover, five days later there were almost no foci in such cells (Figure 5a). The results of immunofluorescent analysis were additionally verified by the FACS data, i.e., there was a significant increase in fluorescence intensity of γH2AX positive cells just after the oxidative stress, while in cells pretreated with DFO it was slightly higher than in the control cells (Figure 5b,c). Thus, DFO pretreatment was sufficient to prevent DNA damage caused by the subsequent H_2_O_2_ addition.

Being a rather large molecule, DFO enters into the cells via endocytosis and accumulates in lysosomes [27,28,29,30]. Earlier it was shown that iron from lysosomes can relocate to the nucleus, where it participates in the Fenton reaction, thus forming extremely reactive hydroxyl radicals [24,28]. Hydroxyl radicals are considered to be the most likely candidates for a rapid DNA damage upon oxidative stress [24,35]. Therefore, we speculated that DNA protective effect of DFO may be related exactly on the prevention hydroxyl radicals’ formation due to intralysosomal iron chelation. To test this suggestion, we utilized dimethyl sulfoxide (DMSO), a well-known solvent that has been shown to act as a hydroxyl radical scavenger [36]. In particular, we performed oxidative stress in a medium without or with 5% DMSO and estimated fluorescent signal using antibodies against γH2AX. Of note, DMSO significantly quenched the fluorescent signal in hMESCs treated with H_2_O_2_, clearly indicating the predominant DNA-damaging role of hydroxyl radicals (Figure 5c and Supplementary Figure S2). At the same time, DMSO had no effect on DFO-pretreated cells exposed to peroxide (Figure 5c), which means that iron chelation with DFO indeed may prevent the formation of hydroxyl radicals and protect DNA from oxidative damage in H_2_O_2_-treated hMESCs.

### 2.5. DFO Pretreatment Is Able to Restore Mitochondrial Membrane Potential during the Progression of Oxidative Stress-Induced Senescence of hMESCs

**Figure 6:**
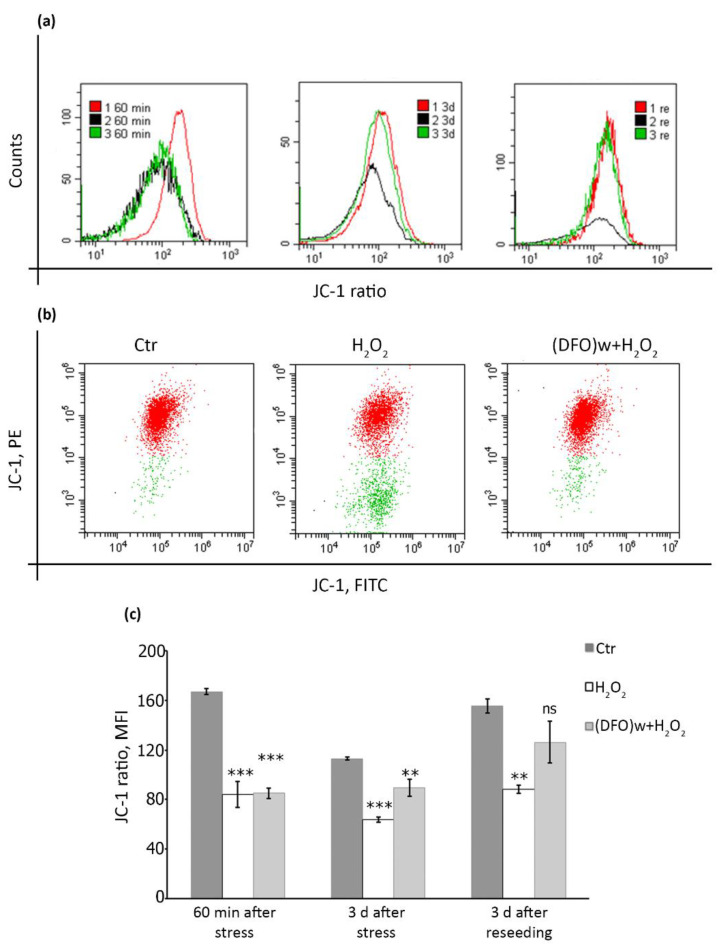
Stress-induced alterations of mitochondrial membrane potential in hMESCs. (**a**) Histograms of JC-1 ratio assayed by FACS; cells were analyzed directly after stress (left panel), three days after (middle panel) and after reseeding (right panel); 1–untreated cells (Ctr), 2–cells exposed to 200 µM of H_2_O_2_ for 1 h, 3–cells pretreated with 1mM of DFO for 2 h, washed and exposed to 200 µM of H_2_O_2_ for 1 h; (**b**) representative dot plots illustrate the dynamics of JC-1 forms’ redistribution (3 d after the treatments): aggregative (red) and monomeric (green), which depends on the mitochondrial membrane potential; (**c**) the same data as in (**a**), depicted graphically; data are shown as Mean ± SD, *n* = 3; *** *p* < 0.005, ** *p* < 0.01.

One of the crucial intracellular alterations sustaining persistent DNA damage during cell senescence is mitochondrial dysfunction. Impaired mitochondrial functioning mediates both elevated ROS generation responsible for the maintenance of the irreversible cycle arrest via the constant DNA damage and impaired oxidative phosphorylation resulting in reduced ATP levels [37]. Thus, we studied the effects of DFO pretreatment on mitochondrial functioning in hMESCs. Mitochondrial health depends on the electrochemical gradient of its inner membrane, which can be assessed using ratiometric lipophilic permeate dye JC-1. Penetrating into healthy intact cells, JC-1 forms so-called J-aggregates with red fluorescence. Since the dye is potential-sensitive, a decrease in mitochondrial membrane potential (MMP) leads to the appearance of the monomeric form of JC-1 with green fluorescence. The ratio of the red and green fluorescence of the dye is a measure of the MMP [38]. Since oxidative stress can itself lead to a rapid drop of MMP [39], we selected the following time points for investigation: (1) immediately after the oxidative stress; (2) three days later, when senescence was already initiated; (3) after cell reseeding and additional cultivation, when senescence was fully developed. As expected, H_2_O_2_ addition led to a rapid drop of MMP in hMESCs, indicating membrane depolarization (Figure 6a–c). Furthermore, MMP remained reduced in H_2_O_2_-treated cells during senescence initiation and progression (Figure 6a,c). Interestingly, DFO pretreatment was not able to prevent the initial drop of MMP caused directly by the oxidizer supplementation (Figure 6a–c). However, in DFO-pretreated hMESCs MMP gradually restored almost to the control level in a delayed time points, demonstrating that DFO pretreatment may prevent the formation of persistent DNA damage foci during hMESCs senescence progression partially by restoring the mitochondrial function (Figure 6a,c).

### 2.6. DFO Causes a Significant Accumulation of HIF-1α in hMESCs

**Figure 7:**
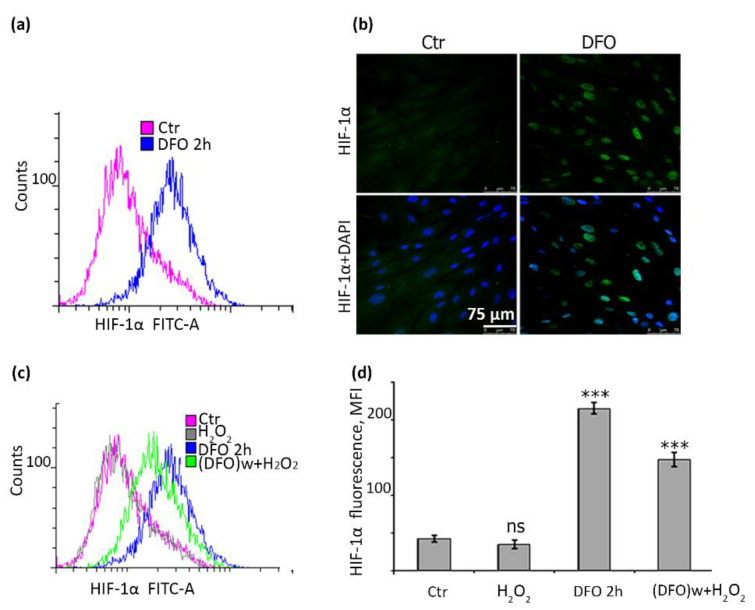
DFO pretreatment induced HIF-1α accumulation both in control and H_2_O_2_-treated hMESCs. Cells were cultured in the presence of 1 mM DFO for 2 h, stained with anti-HIF-1α antibodies and assayed by FACS (**a**) and by immunofluorescence (**b**); (**c**) FACS analysis of cells stained with anti-HIF-1α antibodies: untreated (ctr), H_2_O_2_-treated for 1 h (H_2_O_2_), DFO-treated for 2 h (DFO 2 h), DFO-pretreated for 2 h, washed and exposed to 200 μM H_2_O_2_ for 1 h ((DFO)w+H_2_O_2_); the same data is shown graphically (**d**). Data are shown as Mean ± SD, *n* = 3; *** *p* < 0.005; immunofluorescence 100× magnification; scale bar = 75 µm.

It is known that DFO not only chelates free iron and prevents Fenton reaction, but also that it can bind to ferrous iron at the active centers of HIF prolyl hydroxylases and thereby induce HIF-1α accumulation [40]. Indeed, we revealed that DFO itself was able to induce more than a four-fold increase in cell fluorescence, indicating accumulation of HIF-1α (Figure 7a). Immunofluorescent analysis also confirmed a marked nuclear accumulation of protein upon DFO supplementation (Figure 7b). HIF-1α was previously proved to have a role in preventing senescence at least under hypoxic conditions [41]. Therefore, we next tested whether DFO-protective effects against H_2_O_2_-induced senescence might correlate with the accumulation of HIF-1α in hMESCs. Importantly, DFO-induced HIF-1α accumulation sustained even upon subsequent H_2_O_2_-treatment (Figure 7c,d), which could serve as an additional barrier against oxidative stress-induced senescence of hMESCs.

## 3. Discussion

Within the present study we tried to investigate the protective effect of DFO on the MSCs cultures exposed to sublethal oxidative stress with the aim to elaborate a new pre-conditioning strategy that can be applied during MSCs transplantation to alleviate negative impact of the oxidative microenvironment in the damaged region on the MSCs functioning. One of the well-known reactions of MSCs induced by the oxidative stress is senescence [14,15,18]. To this end, we utilized the model of oxidative stress-induced senescence of hMESCs designed and studied in detail in our previous investigations [14,26], and tested the possibility of DFO to prevent its progression. DFO belongs to bacterial siderophores—compounds that possess a high affinity for iron [42,43]. Currently, there are several evidences that support the application of DFO with regard to MSCs. For instance, a DFO pretreatment technique was shown to enhance the migratory ability of MSCs, which can be useful for further directed homing [44,45]. Additionally, DFO-treated MSCs were found to be more resistant towards H_2_O_2_-induced cell death [13,46]. However, the data regarding DFO administration as a strategy to prevent acute stress-induced senescence of MSCs are lacking.

Since there are several contradictory evidences of DFO effects on cell functioning, the essential goal of the present study was to optimize the treatment scheme of hMESCs with DFO. In earlier studies the protective role of DFO against senescent-like growth arrest was shown in human fibroblasts exposed to oxidative stress [37,47]. Pretreatment of lymphoblastoid T cells 1301 with DFO was sufficient to protect cells from oxidative DNA damage [24]. On the contrary, several studies using different cell models have demonstrated that DFO can itself induce senescence in mammalian cells [48,49,50,51,52]. Therefore, DFO can cause both beneficial and damaging effects on cultured cells, probably depending on its concentration and application mode. Within the present study we revealed that pretreatment with 1 mM of DFO for 2 h was sufficient to achieve the maximal protective effect against H_2_O_2_-induced growth arrest. Moreover, upon selected treatment conditions DFO was able to prevent oxidative stress-induced senescence of hMESCs. Along with the preserved proliferative potential, DFO supplementation averted cell hypertrophy, lipofuscine and ROS accumulation, as well as SA-β-Gal activation. Of note, the characteristics of DFO pretreated hMESCs remained indistinguishable from the control ones long after the oxidative stress and even after additional reseeding, indicating that the designed DFO treatment scheme is sufficient to fully prevent any undesirable consequences associated with oxidative stress-induced senescence of hMESCs. At the same time, cell senescence is considered to be crucial tumor-suppressor mechanism that prevents propagation of the cells bearing damages [34]. In this regard, once rescued from the oxidative stress, DFO pretreated hMESCs should necessarily preserve this stress reaction; otherwise, such cells being transplanted might promote transformation and cancer progression. Indeed, we demonstrated that repeated oxidative stress resulted in senescence induction in hMESCs that previously overcame senescence due to DFO pretreatment. Together, we can state that the designed DFO application scheme is an effective and safe approach to prevent oxidative stress-induced senescence of hMESCs. 

We further tried to uncover possible mechanisms of the protective DFO action. DNA damage is an extremely important and almost immediate intracellular reaction on the oxidative stress. Previously, we detected the appearance of γ-H2AX foci in hMESCs several minutes after H_2_O_2_ addition, with the number of foci gradually increasing, reaching the maximum amount at the end of the treatment [26]. H_2_O_2_-induced DNA damage led to the activation of DNA damage response (DDR) and p53/p21/Rb pathway resulting in a rapid cell cycle block. Though the main part of these foci was efficiently repaired shortly after the end of the treatment, several large foci persisted in senescent cells long after DNA damaging stress. Such persistent large foci are the common feature of senescent cells and are likely to appear and maintain as a result of impaired mitochondrial functioning, increased ROS production and reduced efficiency of the repair systems during senescence progression [26,53]. Importantly, DFO pretreatment almost completely prevented formation of both types of foci in hMESCs—small foci induced directly by H_2_O_2_ and large ones formed during senescence development. Hydroxyl radicals are believed to be the most likely candidates for DNA damage during the oxidative stress [24,35]. In line with this notion, additional supplementation of H_2_O_2_-treated hMESCs with DMSO (an antioxidant proved to scavenge hydroxyl radicals [54], reduced formation of DNA damage foci. Interestingly, DFO itself caused even more pronounced protective effects against DNA damage, and DMSO addition did not induce any extra protection. Based on that, we can speculate that the protective effects of DFO against the oxidative stress-induced DNA damage in hMESCs are partially related to the efficient prevention of hydroxyl radical formation.

Being a rather small molecule, H_2_O_2_ can easily penetrate through cellular and intracellular membranes and cause a rapid drop of mitochondrial membrane potential (MMP) [39]. In line with this notion, we revealed significant MMP loss just after H_2_O_2_ treatment. Importantly, MMP remained reduced in hMESCs as senescence progressed. Decreased MMP is one of the hallmarks of senescent cells, which reflect impaired oxidative phosphorylation and overall mitochondrial dysfunction [53]. Previously, it was shown that pretreatment of Jurkat cells with DFO followed by the oxidative stress was sufficient to rescue mitochondria from oxidative damage and MMP reduction [30]. Unexpectedly, in our experimental system DFO pretreatment was not able to protect hMESCs from the rapid MMP loss upon H_2_O_2_ treatment. The observed difference might be due to the various approaches performed to generate oxidative stress. While in the earlier study the authors utilized cultivation in presence of glucose oxidase in the culture medium to generate slowly developing oxidative stress [30], we used pulse exogenous H_2_O_2_ supplementation. The inner mitochondrial membrane is permeable to small neutral molecules, thus exogenously added H_2_O_2_ can rapidly permeate inside mitochondrion and cause drop of MMP [55]. Contrarily to H_2_O_2_, DFO is fairly large hydrophilic molecule that enters into the cells through liquid-phase endocytosis, and therefore hardly can achieve mitochondrion [27]. Based on the different dynamics of cell entrance, in our experimental conditions DFO was not able to prevent the Fenton reaction inside the mitochondrial matrix and to protect mitochondria from the oxidative damage. Nevertheless, despite the initial MMP loss upon the oxidative stress, further it restored in DFO-pretreated hMESCs reaching the same level as in control ones, while in H_2_O_2_-treated cells it remained reduced. These data allowed suggesting that the mechanisms of the protective DFO action differ from the direct neutralization of H_2_O_2_ molecules, and that cells that escaped senescence due to DFO protection, are able to restore normal physiology. 

An additional mechanism of the protective DFO action against oxidative stress-induced senescence of hMESCs might be associated with accumulation and stabilization of HIF-1α. The well-described physiological HIF-1α inducer is hypoxia. There are clear evidences in favor of the protective role of hypoxia against senescence [56,57,58,59]. For example, it was shown that cells undergoing hypoxia (1–1.5% oxygen) had a greater number of doublings and an increase in life expectancy compared to normoxia (20–21% oxygen) [58,60]. DFO is often called hypoxia mimetic for its ability to induce HIF-1α accumulation [61,62,63]. According to our data, DFO pretreatment indeed led to HIF-1α accumulation in H_2_O_2_-treated hMESCs, which can be considered as an additional mechanism to protect cells from proliferative delay and senescence development. To sum up, within the present study we designed effective and safe protocol based on the single dose DFO pretreatment that will allow preventing stress-induced senescence of MSCs upon transplantation. The developed strategy could improve regenerative capacity of cells to be transplanted. Bearing in mind that the regenerative capacity of transplanted MSCs is mediated primarily via paracrine activity, together with the fact that DFO can modulate MSCs secretome, it would be of a particular interest to compare paracrine activity of untreated and DFO-pretreated hMESCs.

## 4. Materials and Methods

### 4.1. Cell Culture

All experiments were performed on human endometrial stem cells (line 2304), which were isolated, characterized and kindly given to us by Zemelko with co-authors [6]. Cells were cultured in DMEM/F12 (Thermo Fisher Scientific, Waltham, MA, USA) medium supplemented with 10% fetal bovine serum (Life Technologies, Carlsbad, CA, USA), 1% glutamax (Thermo Fisher Scientific, Waltham, MA, USA) and 1% penicillin-streptomycin (Thermo Fisher Scientific, Waltham, MA, USA) at 37 °C in 5% CO_2_ atmosphere. To avoid overlapping of cell responses because of replicative and induced senescence, we used cells that pass no more than ten passages. All experiments performed on human cells were carried out in accordance with the standards of the Helsinki Declaration (1989) that were accepted and claimed by the Ethics Commission of the Institute of Cytology of RAS. 

### 4.2. Experiment Design and Cell Treatments

Previously, we have already used hMESCs for the development of stress induced premature senescence (SIPS) model [14,26]. According to the elaborated protocol, cells were seeded with a density 6 × 10^5^ cells/mL (about 70–80% of monolayer) cells per Petri dish of 35 mm diameter. Deferoxamine mesylate (Sigma-Aldrich, St. Louis, MO, USA) was prepared immediately before use as a 10-fold solution in serum-free medium. In one series of experiments, we varied its concentration from 0.01 to 10 mM in order to select the optimal concentration that would provide the best protective effect from the cessation of cell growth caused by H_2_O_2_. In other series of experiments, cells were pretreated with 1 mM DFO for 2 h, after which they were washed twice with serum-free medium. Then SIPS was induced with 200 μM of H_2_O_2_ applied for 1 h (a 10-fold solution in PBS was prepared from a 35% stock solution; Sigma-Aldrich, St. Louis, MO, USA). The peroxide dose was about 2.0 pmol per cell. After H_2_O_2_ exposure, cells were washed with PBS and analyzed either immediately or at selected time points after prolonged cultivation, depending on the objectives of the study. Before reseeding, the number of cells was calculated and aligned according to the number of cells treated with H_2_O_2_ only. In some experiments, 5% dimethyl sulfoxide (DMSO; Sigma-Aldrich, St. Louis, MO, USA) was added before the treatment with H_2_O_2_. 

### 4.3. Flow Cytometry Assay

Samples were analyzed with CytoFLEX flow cytometer (Beckman Coulter, Brea, CA, USA) or CytoFLEX S flow cytometer (Beckman Coulter, USA). Data assayed by CytExpert software (versions 1.2 and 2.0, Brea, CA, USA) are presented as diagrams, dotplots and histograms. 

#### 4.3.1. Viability, Autofluorescence, Forward Scattering Assay

Before the analysis, cells were harvested by trypsinization and suspended in culture medium. A total of 50 μg/mL of propidium iodide (PI) was added to the cell suspension and gently mixed for 30 s. At least 3000 events were usually collected as the main cell population. Raw data were gated by elimination of debris identified as FSC-A low and SSC-A low (Supplementary Figure S3). Representative PI vs FSC dotplots allowed us to distinguish between PI-negative “live” cells and PI-positive “dead” cells, while simultaneously evaluating the number of cells, the mean value of autofluorescence (AF), which reflects the presence of lipofuscin, and the mean value of forward scattering (FS), which correlates with the cell size. 

#### 4.3.2. Cell Cycle Analysis

To the cells harvested by trypsinization, 250 μg/mL RNase (Sigma-Aldrich, St. Louis, MO, USA), 300 μg/mL saponin (Alfa Aesar, Karlsruhe, Germany) and 50 μg/mL PI (Sigma-Aldrich, St. Louis, MO, USA) were added and incubated for 1 h in the dark at RT. At least 15,000 stained cells were collected and analyzed using flow cytometry. 

#### 4.3.3. Qualification of Intracellular ROS, Mitochondrial Mass, Mitochondrial Membrane Potential

To determine the ROS and mitochondrial mass, the monolayer of cells was incubated in a serum-free medium with dichlorodihydrofluorescein diacetate (H2DCFDA) and nonyl acridine orange (NAO), respectively. A cell suspension for FACS analysis was prepared as described previously [26].

The mitochondrial membrane potential (MMP) was measured after staining with the ratiometric dye 5,5,6,6′-tetrachloro-1,1′,3,3′ tetraethylbenzimi-dazoylcarbocyanine iodide (JC-1; Invitrogen, Carlsbad, CA, USA). The lipophilic dye JC-1 accumulates inside the mitochondria in the form of so-called J-aggregates (red fluorescence), which can be reversibly converted to the monomeric form of the dye (green fluorescence), which depends on the polarization/depolarization of the mitochondrial membrane. Therefore, the red/green fluorescence ratio is an important characteristic of mitochondrial health. The ratio parameter (PE/FITC ratio) was defined in the measurement protocol using the “Set customized parameter” option. The staining procedure was carried out in accordance with the described protocol [38]. For this, 2 μM JC-1 was added to cells suspended in a preheated full growth medium and placed in an incubator at 37 °С with 5% СО_2_ for 45–60 min. A 10-fold solution of JC-1 in PBS was prepared just before adding. The cells are then washed with excess PBS, pelleted by centrifugation, suspended in PBS, and MMP was evaluated by measuring the JC-1 ratio using flow cytometry. CytoFLEX S cytometer allows simultaneous exciting the JC-1 dye by different lasers (488 nm and 561 nm) and thus, avoiding the additional compensation settings. 

#### 4.3.4. Determination of SA-β-Gal activity

Cells expressing senescent-associated β-galactosidase were detected with senescence β-galactosidase staining kit (Cell Signaling Technology, Beverly, MA, USA, #9860) according to manufacturer’s instructions. The kit detects β-galactosidase activity at pH 6.0 in cultured cells which is present only in senescent cells and is not found in pre-senescent, quiescent or immortal cells. 

#### 4.3.5. Determination of DNA Damage, HIF-1α Accumulation and Cyclin D1

Cells detached from Petri dishes were washed twice with PBS, calculated and adjusted to 1 × 10^6^/mL; 2 × 10^5^/mL cells of each sample were fixed, permeabilized using True-Nuclear™ Transcription Factor Buffer Set (BioLegend, San Diego, CA, USA) and stained by relevant antibodies.

To detect single-strand DNA breaks, hMESCs were incubated with anti-γH2AX antibodies (1:200; Abcam, Cambridge, UK) overnight at +4 °C in the dark. After incubation, the cells were washed with PBS and suspended in a perm buffer for staining with secondary antibodies (1:500; GAM Alexa Fluor^®^ 488) for 1 h at room temperature in the dark; 1 μg/mL DAPI was also added for staining cellular DNA. After incubation, the cells were washed and suspended in PBS. 

To detect the accumulation of HIF-1α, fixed and permeable hMESCs were stained according to the protocol described above using first antibodies (1:2000; Cell Signaling Technology, Beverly, MA, USA) and second ones (1:1000; GAM Alexa Fluor^®^ 488). The analysis was performed on a CytoFLEX S flow cytometer equipped with 375/488 nm lasers.

Cyclin D1 was identified by direct staining of fixed and permeabilized cells with anti-cyclin D1 antibodies conjugated to Alexa Fluor^®^ 647 (1:50, Abcam). The analysis was performed on a CytoFLEX flow cytometer equipped with lasers with 405/638 nm wavelengths. 

### 4.4. Immunofluorescent Assay

Treated cells grown on coverslips were assayed directly after oxidative stress finished or five days after. hMESCs were fixed with 4% formaldehyde (15 min), permeabilized with 0.1% Triton X-100 (10 min) and blocked with 1% BSA (1 h). Cells were incubated with primary antibodies‒a rabbit polyclonal antibodies against Phospho-Histone γH2A.X (Ser139, 20E3, Cell Signaling), overnight at 4 °C and secondary antibodies‒Goat anti-Rabbit IgG (H + L) Cross-Adsorbed (Alexa Fluor 568, Invitrogen) for 1 h at room temperature after extensive washing with PBS/0.1% Tween 20 between each step. The slides were counterstained with 1 μg/mL DAPI (Sigma) and mounted using 2% propyl gallate. The coverslips were imaged with laser-scanning microscope Leica TCS SL performed using ImageJ software (US National Institutes of Health, Bethesda, MD, USA).

For HIF-1α detection cells grown on cover slips were took out only directly after stress finished/Cells were fixed with 4% formaldehyde (15 min), permeabilized with 0.1% Triton X-100 (10 min) and blocked with 1% BSA (1 h). The cells were incubated with primary antibodies—mouse monoclonal antibodies anti-HIF-1α (1:800, Cell Signaling)—overnight at 4 °C, and secondary antibodies—Alexa Fluor 488 goat anti-mouse (GAR)—for 1 h at room temperature after extensive washing with PBS/0.1% Tween 20 between each step. The slides were counterstained with 1 μg/mL DAPI (Sigma) and mounted using 2% propyl gallate. A Zeiss Axiovert 200M fluorescence microscope (Carl Zeiss, Jena, Germany) equipped with a digital camera DFC 420C (Leica, Wetzlar, Germany) utilizing Adobe Photoshop software was used to view and acquire images. 

### 4.5. Live Cell Imaging and Confocal Microscopy Detection of Lipofuscin within Lysosomal Compartment

The live-cell imaging was conducted using Leica TCS SP5 inverted laser scanning confocal microscope (Germany) and a thermostatic chamber, using an oil immersion objective of 40×, 1.25 NA. The base plane of the cell was imaged with the scanning frequency 400–700 Hz and image size of 1024 × 1024 pixels.

Untreated, H_2_O_2_ treated only, and pretreated with DFO then exposed with peroxide cells were cultured for five days in 33 mm Petri dishes. All cells were then reseeded on 4-well μ-slides (Ibidi, Denmark) for real-time imaging. Before seeding, the number of cells was calculated and aligned according to the number of cells treated with H_2_O_2_. After culturing for an additional two days, 50 nM vital dye LysoTracker Red was added to the cells and incubated for 30 min at 37 °C (Molecular Probes, Eugene, OR, USA). Then medium was replaced to Fluoro Brite DMEM (Gibco^®^ FluoroBrite™ DMEM, A 18967-01) medium for the enhancement of fluorescence signal during live-cell imaging and analyzed using a temperature and gas control chamber (25 °C and 5% CO_2_) of the microscope. LysoTracker Red was excited at 543 nm and recorded in the range 565–680 nm. Since lipofuscin has a broad autofluorescence band, lipofuscin granules can be observed using confocal microscopy using excitation light of different wavelengths (ultraviolet 330–380 nm, blue 450–490 nm and green 510–560 nm) as well as 420, 520 and 590 nm barrier filters, respectively.

Data were collected by Leica software as raw lif files and transferred as a series of tiff files for further analysis. 

### 4.6. Statistical Analyses

Unless otherwise indicated, all quantitative data are shown as M ± S.D. To get significance in the difference between two groups Students t-test was applied. For multiple comparisons between groups, ANOVA with Tukey HSD was used. Statistical analysis was performed using R software.

## 5. Conclusions

We have proposed an approach using pretreatment of hMESCs with DFO, which allows cells to avoid the induction and progression of senescence caused by oxidative stress. Due to the antioxidant and hypoximimetic capacity of DFO, cells adequately respond to oxidative stress and retain their native properties.

## Data Availability

The raw data to all Figures are available online at https://www.mdpi.com/article/10.3390/ijms22116035/s1 in the Supplementary File.

## Supplementary Materials

The following are available online at https://www.mdpi.com/article/10.3390/ijms22116035/s1.

## References

[1] Fernández Vallone V.B., Romaniuk M.A., Choi H., Labovsky V., Otaegui J., Chasseing N.A. (2013). Mesenchymal stem cells and their use in therapy: What has been achieved?. Differentiation.

[2] Friedenstein A.J., Petrakova K.V., Kurolesova A.I., Frolova G.P. (1968). Heterotopic of bone marrow. Analysis of precursor cells for osteogenic and hematopoietic tissues. Transplantation.

[3] Berebichez-Fridman R., Montero-Olvera P.R. (2018). Sources and Clinical Applications of Mesenchymal Stem Cells: State-of-the-art review. Sultan Qaboos Univ. Med. J..

[4] Gargett C.E., Masuda H. (2010). Adult stem cells in the endometrium. Mol. Hum. Reprod..

[5] Xu Y., Zhu H., Zhao D., Tan J. (2015). Endometrial stem cells: Clinical application and pathological roles. Int. J. Clin. Exp. Med..

[6] Zemelko V.I., Grinchuk T.M., Domnina A.P., Artzibasheva I.V., Zenin V.V., Kirsanov A.A., Bichevaia N.K., Korsak V.S., Nikolsky N.N. (2012). Multipotent mesenchymal stem cells of desquamated endometrium: Isolation, characterization, and application as a feeder layer for maintenance of human embryonic stem cells. Cell Tissue Biol..

[7] Ulrich D., Muralitharan R., Gargett C.E. (2013). Toward the use of endometrial and menstrual blood mesenchymal stem cells for cell-based therapies. Expert. Opin. Biol. Ther..

[8] Geng Y.J. (2003). Molecular mechanisms for cardiovascular stem cell apoptosis and growth in the hearts with atherosclerotic coronary disease and ischemic heart failure. Ann. N. Y. Acad. Sci..

[9] Lee K.A., Shim W., Paik M.J., Lee S.C., Shin J.Y., Ahn Y.H., Park K., Kim J.H., Choi S., Lee G. (2009). Analysis of changes in the viability and gene expression profiles of human mesenchymal stromal cells over time. Cytotherapy.

[10] Liu X.B., Jiang J., Gui C., Hu X.Y., Xiang M.X., Wang J.A. (2008). Angiopoietin-1 protects mesenchymal stem cells against serum deprivation and hypoxia-induced apoptosis through the PI3K/Akt pathway. Acta Pharmacol. Sin..

[11] Leibacher J., Dauber K., Ehser S., Brixner V., Kollar K., Vogel A., Spohn G., Schäfer R., Seifried E., Henschler R. (2017). Human mesenchymal stromal cells undergo apoptosis and fragmentation after intravenous application in immune-competent mice. Cytotherapy.

[12] Lee S., Choi E., Cha M.J., Hwang K.C. (2015). Cell adhesion and long-term survival of transplanted mesenchymal stem cells: A prerequisite for cell therapy. Oxidative Med. Cell. Longev..

[13] Khoshlahni N., Sagha M., Mirzapour T., Zarif M.N., Mohammadzadeh-Vardin M. (2020). Iron depletion with deferoxamine protects bone marrow-derived mesenchymal stem cells against oxidative stress-induced apoptosis. Cell Stress Chaperones.

[14] Burova E., Borodkina A., Shatrova A., Nikolsky N. (2013). Sublethal oxidative stress induces the premature senescence of human mesenchymal stem cells derived from endometrium. Oxidative Med. Cell. Longev..

[15] Alekseenko L.L., Zemelko V.I., Domnina A.P., Lyublinskaya O.G., Zenin V.V., Pugovkina N.A., Kozhukharova I.V., Borodkina A.V., Grinchuk T.M., Fridlyanskaya I.I. (2014). Sublethal heat shock induces premature senescence rather than apoptosis in human mesenchymal stem cells. Cell Stress Chaperones.

[16] Liu J., Ding Y., Liu Z., Liang X. (2020). Senescence in mesenchymal stem cells: Functional alterations, molecular mechanisms, and rejuvenation strategies. Front. Cell Dev. Biol..

[17] Domnina A., Ivanova J., Alekseenko L., Kozhukharova I., Borodkina A., Pugovkina N., Smirnova I., Lyublinskaya O., Fridlyanskaya I., Nikolsky N. (2020). Three-Dimensional Compaction Switches Stress Response Programs and Enhances Therapeutic Efficacy of Endometrial Mesenchymal Stem/Stromal Cells. Front. Cell Dev. Biol..

[18] Turinetto V., Vitale E., Giachino C. (2016). Senescence in human mesenchymal stem cells: Functional changes and implications in stem cell-based therapy. Int. J. Mol. Sci..

[19] Griukova A., Deryabin P., Shatrova A., Burova E., Severino V., Farina A., Nikolsky N., Borodkina A. (2019). Molecular basis of senescence transmitting in the population of human endometrial stromal cells. Aging.

[20] Konala V.B., Mamidi M.K., Bhonde R., Das A.K., Pochampally R., Pal R. (2016). The current landscape of the mesenchymal stromal cell secretome: A new paradigm for cell-free regeneration. Cytotherapy.

[21] Tran C., Damaser M.S. (2015). Stem cells as drug delivery methods: Application of stem cell secretome for regeneration. Adv. Drug Deliv. Rev..

[22] Vizoso F.J., Eiro N., Cid S., Schneider J., Perez-Fernandez R. (2017). Mesenchymal Stem Cell Secretome: Toward Cell-Free Therapeutic Strategies in Regenerative Medicine. Int. J. Mol. Sci..

[23] Persson H.L., Yu Z., Tirosh O., Eaton J.W., Brunk U.T. (2003). Prevention of oxidant-induced cell death by lysosomotropic iron chelators. Free Radic. Biol. Med..

[24] Kurz T., Leake A., Von Zglinicki T., Brunk U.T. (2004). Relocalized redox-active lysosomal iron is an important mediator of oxidative-stress-induced DNA damage. Biochem. J..

[25] Lloyd J.B., Cable H., Rice-Evans C. (1991). Evidence that desferrioxamine cannot enter cells by passive diffusion. Biochem. Pharmacol..

[26] Borodkina A., Shatrova A., Abushik P., Nikolsky N., Burova E. (2014). Interaction between ROS dependent DNA damage, mitochondria and p38 MAPK underlies senescence of human adult stem cells. Aging.

[27] Doulias P.T., Christoforidis S., Brunk U.T., Galaris D. (2003). Endosomal and lysosomal effects of desferrioxamine: Protection of HeLa cells from hydrogen peroxide-induced DNA damage and induction of cell-cycle arrest. Free Radic. Biol. Med..

[28] Kurz T., Gustafsson B., Brunk U.T. (2006). Intralysosomal iron chelation protects against oxidative stress-induced cellular damage. FEBS J..

[29] Barbouti A., Doulias P.T., Zhu B.Z., Frei B., Galaris D. (2001). Intracellular iron, but not copper, plays a critical role in hydrogen peroxide-induced DNA damage. Free Radic. Biol. Med..

[30] Tenopoulou M., Doulias P.T., Barbouti A., Brunk U., Galaris D. (2005). Role of compartmentalized redox-active iron in hydrogen peroxide-induced DNA damage and apoptosis. Biochem. J..

[31] Terman A., Dalen H., Eaton J.W., Neuzil J., Brunk U.T. (2004). Aging of cardiac myocytes in culture: Oxidative stress, lipofuscin accumulation, and mitochondrial turnover. Ann. N. Y. Acad. Sci..

[32] Bertolo A., Baur M., Guerrero J., Pötzel T., Stoyanov J. (2019). Autofluorescence is a reliable in vitro marker of cellular senescence in human mesenchymal stromal cells. Sci. Rep..

[33] Leontieva O.V., Demidenko Z.N., Blagosklonny M.V. (2013). MEK drives cyclin D1 hyperelevation during geroconversion. Cell Death Differ..

[34] Campisi J. (2001). Cellular senescence as a tumor-suppressor mechanism. Trends Cell Biol..

[35] Chen Q.M., Bartholomew J.C., Campisi J., Acosta M., Reagan J.D., Ames B.N. (1998). Molecular analysis of H_2_O_2_-induced senescent-like growth arrest in normal human fibroblasts: p53 and Rb control G1 arrest but not cell replication. Biochem. J..

[36] Sanmartín-Suárez C., Soto-Otero R., Sánchez-Sellero I., Méndez-Álvarez E. (2011). Antioxidant properties of dimethyl sulfoxide and its viability as a solvent in the evaluation of neuroprotective antioxidants. J. Pharmacol. Toxicol. Methods.

[37] Shmulevich R., Krizhanovsky V. (2021). Cell Senescence, DNA Damage, and Metabolism. Antioxid. Redox Signal..

[38] Sivandzade F., Bhalerao A., Cucullo L. (2019). Analysis of the mitochondrial membrane potential using the cationic JC-1 dye as a sensitive fluorescent probe. Bio Protoc..

[39] Tada-Oikawa S., Oikawa S., Kawanishi M., Yamada M., Kawanishi S. (1999). Generation of hydrogen peroxide precedes loss of mitochondrial membrane potential during DNA alkylation-induced apoptosis. FEBS Lett..

[40] Esfahani M., Karimi F., Afshar S., Niknazar S., Sohrabi S., Najafi R. (2015). Prolyl hydroxylase inhibitors act as agents to enhance the efficiency of cell therapy. Expert. Opin. Biol. Ther..

[41] Welford S.M., Bedogni B., Gradin K., Poellinger L., Broome Powell M., Giaccia A.J. (2006). HIF1alpha delays premature senescence through the activation of MIF. Genes Dev..

[42] Miethke M., Marahiel M.A. (2007). Siderophore-based iron acquisition and pathogen control. Microbiol. Mol. Biol. Rev..

[43] Franchini M., Gandini G., de Gironcoli M., Vassanelli A., Borgna-Pignatti C., Aprili G. (2000). Safety and efficacy of subcutaneous bolus injection of deferoxamine in adult patients with iron overload. Blood.

[44] Najafi R., Sharifi A.M. (2013). Deferoxamine preconditioning potentiates mesenchymal stem cell homing in vitro and in streptozotocin-diabetic rats. Expert. Opin. Biol. Ther..

[45] Peyvandi A.A., Abbaszadeh H.A., Roozbahany N.A., Pourbakht A., Khoshsirat S., Niri H.H., Peyvandi H., Niknazar S. (2018). Deferoxamine promotes mesenchymal stem cell homing in noise-induced injured cochlea through PI3K/AKT pathway. Cell Prolif..

[46] Nouri F., Salehinejad P., Nematollahi-Mahani S.N., Kamarul T., Zarrindast M.R., Sharifi A.M. (2016). Deferoxamine preconditioning of neural-like cells derived from human Wharton’s Jelly mesenchymal stem cells as a strategy to promote their tolerance and therapeutic potential: An in vitro study. Cell. Mol. Neurobiol..

[47] Chen Q., Ames B.N. (1994). Senescence-like growth arrest induced by hydrogen peroxide in human diploid fibroblast F65 cells. Proc. Natl. Acad. Sci. USA.

[48] Yoon Y.S., Cho H., Lee J.H., Yoon G. (2004). Mitochondrial dysfunction via disruption of complex II activity during iron chelation-induced senescence-like growth arrest of Chang cells. Ann. N. Y. Acad. Sci..

[49] Yoon G., Kim H.J., Yoon Y.S., Cho H., Lim I.K., Lee J.H. (2002). Iron chelation-induced senescence-like growth arrest in hepatocyte cell lines: Association of transforming growth factor beta1 (TGF-beta1)-mediated p27Kip1 expression. Biochem. J..

[50] Coleman P.R., Chang G., Hutas G., Grimshaw M., Vadas M.A., Gamble J.R. (2013). Age-associated stresses induce an anti-inflammatory senescent phenotype in endothelial cells. Aging.

[51] Zeng H.L., Zhong Q., Qin Y.L., Bu Q.Q., Han X.A., Jia H.T., Liu H.W. (2011). Hypoxia-mimetic agents inhibit proliferation and alter the morphology of human umbilical cord-derived mesenchymal stem cells. BMC Cell Biol..

[52] Yoon Y.S., Byun H.O., Cho H., Kim B.K., Yoon G. (2003). Complex II defect via down-regulation of iron-sulfur subunit induces mitochondrial dysfunction and cell cycle delay in iron chelation-induced senescence-associated growth arrest. J. Biol. Chem..

[53] Passos J.F., Nelson G., Wang C., Richter T., Simillion C., Proctor C.J., Miwa S., Olijslagers S., Hallinan J., Wipat A. (2010). Feedback between p21 and reactive oxygen production is necessary for cell senescence. Mol. Syst. Biol..

[54] Babbs C.F., Steiner M.G. (1990). Detection and quantitation of hydroxyl radical using dimethyl sulfoxide as molecular probe. Methods Enzymol..

[55] Guo C., Sun L., Chen X., Zhang D. (2013). Oxidative stress, mitochondrial damage and neurodegenerative diseases. Neural Regen. Res..

[56] Kaeberlein M., Kapahi P. (2009). The hypoxic response and aging. Cell Cycle.

[57] Leontieva O.V., Natarajan V., Demidenko Z.N., Burdelya L.G., Gudkov A.V., Blagosklonny M.V. (2012). Hypoxia suppresses conversion from proliferative arrest to cellular senescence. Proc. Natl. Acad. Sci. USA.

[58] Jin Y., Kato T., Furu M., Nasu A., Kajita Y., Mitsui H., Ueda M., Aoyama T., Nakayama T., Nakamura T. (2010). Mesenchymal stem cells cultured under hypoxia escape from senescence via down-regulation of p16 and extracellular signal regulated kinase. Biochem. Biophys. Res. Commun..

[59] Lee J.H., Yoon Y.M., Lee S.H. (2017). Hypoxic preconditioning promotes the bioactivities of mesenchymal stem cells via the HIF-1α-GRP78-Akt axis. Int. J. Mol. Sci..

[60] Alique M., Sánchez-López E., Bodega G., Giannarelli C., Carracedo J., Ramírez R. (2020). Hypoxia-Inducible Factor-1α: The Master Regulator of Endothelial Cell Senescence in Vascular Aging. Cells.

[61] Guo M., Song L.P., Jiang Y., Liu W., Yu Y., Chen G.Q. (2006). Hypoxia-mimetic agents desferrioxamine and cobalt chloride induce leukemic cell apoptosis through different hypoxia-inducible factor-1alpha independent mechanisms. Apoptosis.

[62] Fujisawa K., Takami T., Okada S., Hara K., Matsumoto T., Yamamoto N., Yamasaki T., Sakaida I. (2018). Analysis of metabolomic changes in mesenchymal stem cells on treatment with desferrioxamine as a hypoxia mimetic compared with hypoxic conditions. Stem Cells.

[63] Liu Y., Cui Y., Shi M., Zhang Q., Wang Q., Chen X. (2014). Deferoxamine promotes MDA-MB-231 cell migration and invasion through increased ROS-dependent HIF-1α accumulation. Cell Physiol. Biochem..

