# Supplementary material for: Outcomes of Deferoxamine Action on H2O2-Induced Growth Inhibition and Senescence Progression of Human Endometrial Stem Cells
Source: Int J Mol Sci. 2021 Jun 3;22(11):6035. doi: 10.3390/ijms22116035 (PMC8199751; doi:10.3390/ijms22116035)
Supplement: Supplementary file 1 [file ijms-22-06035-s001.zip › ijms-1192263-SI.pdf]

Supplementary Materials

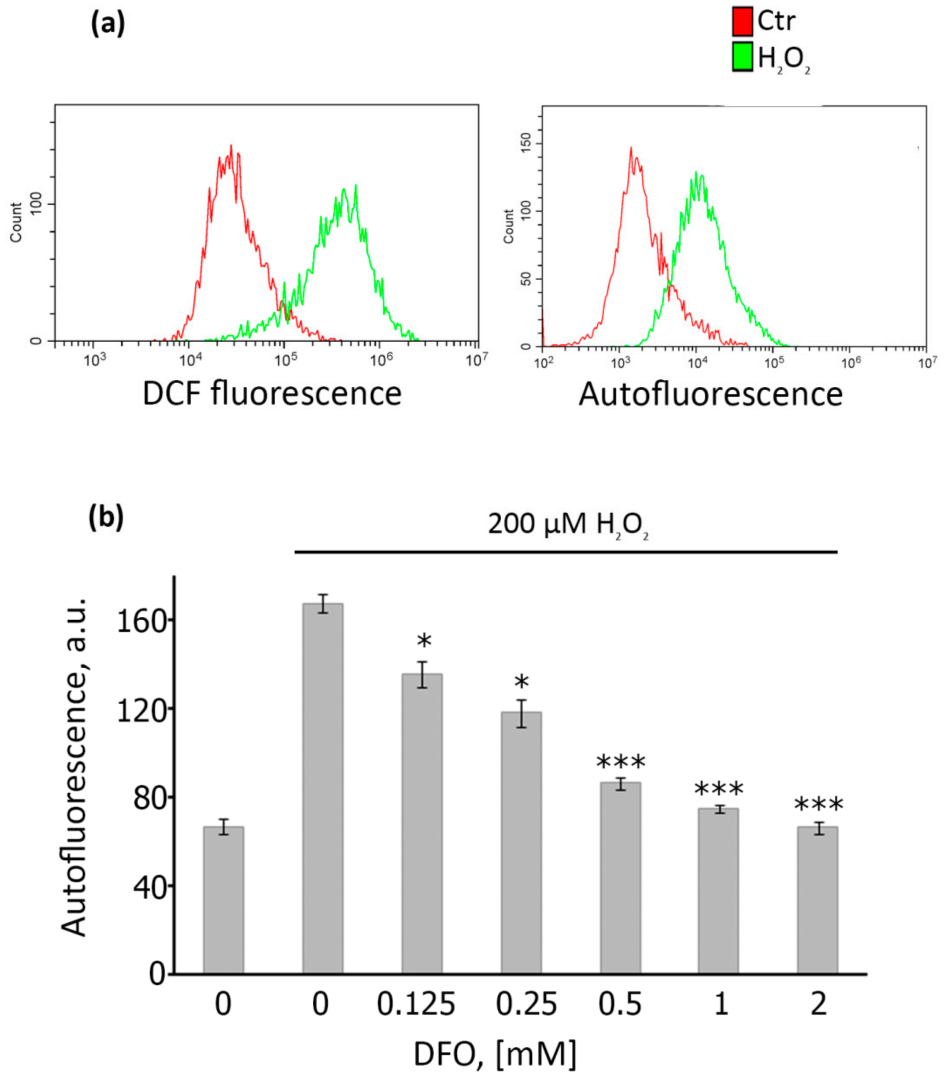

Figure S1. (a) The augmentation in ROS production is accompanied by the accumulation of lipofuscin in hMESCs during the stress-induced premature senescence progression. Cells were exposed to 200 μM of H2O2 for 1 h, washed from peroxide and cultivated for additional five days; FACS histograms of fluorescence intensity of hMESCs are presented – cells were stained with DCF fluorescent probe (on the left) or unstained (on the right). (b) Dose-dependent effect of DFO on stress-induced accumulation of lipofuscin. Cells were pretreated with DFO (DFO concentration varied from 125 μM to 2 mM) for 2 h, then washed from DFO and subjected to oxidative stress with 200 μM of H2O2 for 1 h. Upon completion of oxidative stress, cells were washed from peroxide and cultured in the fresh complete medium for an additional five days. Using FACS, we assessed autofluorescence as a measure of lipofuscin accumulation. Data are shown as Mean ± SD, n=3; ***p<0.005, **p<0.01, *p< 0.05.

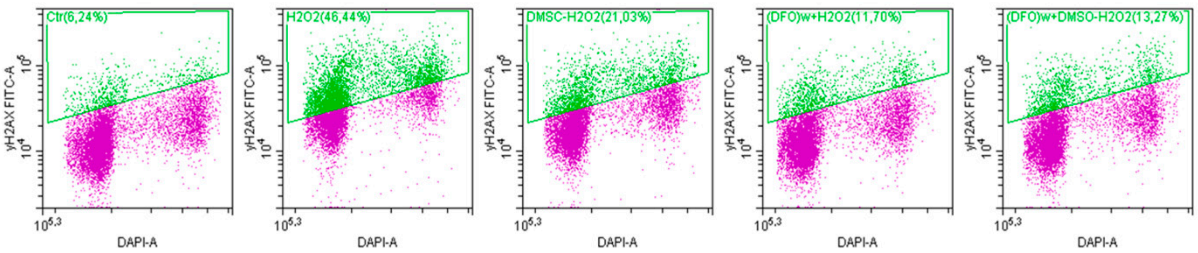

Ctr	H_2O_2	H_2O_2 +DMSO	(DFO)w+H_2O_2	(DFO)w+H_2O_2 +DMSO
6.24%	46.44%	21.03%	11.70%	13.27%

Figure S2. representative FACS dotplots of samples stained with γH2AX antibodies are presented: 1 – untreated cells (Ctr); 2 – cells exposed to 200 μM of H2O2 for 1 h; 3 – cells exposed to 200 μM of H2O2 for 1 h in the medium supplemented with 5% DMSO; 4 - cells pretreated with 1mM of DFO for 2 h, washed and exposed to 200 μM of H2O2 for 1 h; 5 - cells pretreated with 1mM of DFO for 2 h, washed and exposed to 200 μM of H2O2 for 1 h in the medium supplemented with 5% DMSO for 1 h.

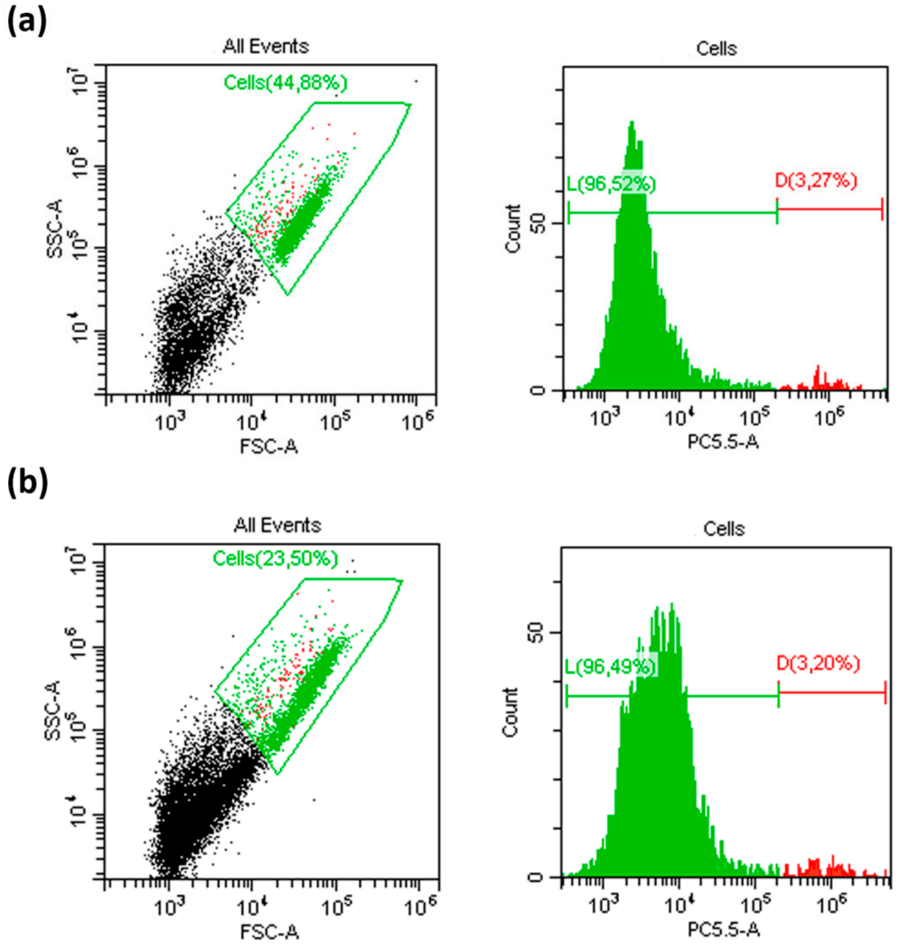

Figure S3. Flow cytometry gating strategy, related to the Figures of the main text. Elimination of debris measurements identified as FSC-A low and SSC-A low and one-parametric histogram reflecting viable and dead cells based on the PI (PC5.5-A) fluorescence intensity provided for young (a) and senescent (b) cells.
